# RNN-AHF Framework: Enhancing Multi-focal Nature of Hypoxic Ischemic Encephalopathy Lesion Region in MRI Image Using Optimized Rough Neural Network Weight and Anti-Homomorphic Filter

**DOI:** 10.2174/0115734056396299250506075133

**Published:** 2025-05-29

**Authors:** M. Thangeswari, R. Muthucumaraswamy, K. Anitha, N.R. Shanker

**Affiliations:** 1 Department of Mathematics, Sri Venkateswara College of Engineering, Affiliated to Anna University, Sriperumbudur, Chennai-602117, Tamil Nadu, India; 2 Department of Mathematics, Amrita School of Engineering, Amrita Vishwa Vidyapeetham, Chennai-601103, Tamil Nadu, India; 3 Department of Computer Science and Engineering, Aalim Muhammed Salegh College of Engineering, Muthapudupet, Chennai-600055, Tamil Nadu, India

**Keywords:** Image enhancement, Anti-homomorphic filter, MRI, Neighborhood rough set, Attribute reduction, Rough neural network

## Abstract

**Introduction:**

Image enhancement of the Hypoxic-Ischemic Encephalopathy (HIE) lesion region in neonatal brain MR images is a challenging task due to the diffuse (*i.e*., multi-focal) nature, small size, and low contrast of the lesions. Classifying the stages of HIE is also difficult because of the unclear boundaries and edges of the lesions, which are dispersed throughout the brain. Moreover, unclear boundaries and edges are due to chemical shifts, partial volume artifacts, and motion artifacts. Further, voxels may reflect signals from adjacent tissues. Existing algorithms perform poorly in HIE lesion enhancement due to artifacts, voxels, and the diffuse nature of the lesion.

**Methods:**

In this paper, we propose a Rough Neural Network and Anti-Homomorphic Filter (RNN-AHF) framework for the enhancement of the HIE lesion region.

**Results:**

The RNN-AHF framework reduces the pixel dimensionality of the feature space, eliminates unnecessary pixels, and preserves essential pixels for lesion enhancement.

**Discussion:**

The RNN efficiently learns and identifies pixel patterns and facilitates adaptive enhancement based on different weights in the neural network. The proposed RNN-AHF framework operates using optimized neural weights and an optimized training function. The hybridization of optimized weights and the training function enhances the lesion region with high contrast while preserving the boundaries and edges.

**Conclusion:**

The proposed RNN-AHF framework achieves a lesion image enhancement and classification accuracy of approximately 93.5%, which is better than traditional algorithms.

## 1 INTRODUCTION

In medical imaging, filters are used to enhance specific regions within images to support accurate diagnosis and prognosis. These filters apply mathematical operations, such as convolution and pixel value modification based on local neighborhoods, to improve image quality. In brain imaging, filtering helps reduce noise and enhances the edges and boundaries of brain tissues. The Homomorphic Filter (HF) is used for the enhancement of tissue regions in brain images. HF adjusts pixel intensity and frequency while preserving tissue structure. HF removes both low and high-frequency noise in brain images. Multiplicative operations in the logarithmic domain enable better visibility of tissue regions in brain images with varying illumination. The non-linear filtering approach of HF reduces noise and enhances the tissues in brain MRI images with artifacts. However, HF faces challenges due to its non-linear nature, necessitating the selection of appropriate parameters, such as scaling factor, filter function, and exponential function for frequency transformation. Selecting the right parameters is complex and time-consuming. Improper parameter selection in HF can lead to over- or under-enhancement of features. Moreover, the computational overhead due to logarithmic transformations reduces enhancement performance.

### 1.1 Problem Statement

In neonatal MR brain images, detecting hypoxic-ischemic encephalopathy lesions is a major problem due to various artifacts, such as partial volume artifacts, chemical shift artifacts, and motion artifacts. Neonates have small brain structures; the number of voxels increases due to mixed signals from adjacent tissues, leading to unenhanced HIE regions. Partial volume artifacts complicate the interpretation of HIE structures. A voxel may contain grey matter, leading to an inaccurate representation of HIE lesions. The chemical shift artifact arises from distinct resonance frequencies of water and fat, resulting in bright and dark bands. These bands lead to misinterpretation of anatomical structures of HIE lesions in neonatal brain images. Neonates are unable to control head and limb movements, as well as physiological motions, such as breathing. These motions lead to artifacts that form ghosting structures in the HIE lesions of neonatal MR images. The motion artifacts cause blurring and distortion in the HIE lesion region, leading to misinterpretation of the anatomical structure of the HIE lesion region and resulting in inaccurate diagnosis of HIE lesions. To solve this problem, an efficient filter for the classification of HIE lesions and efficient Deep Learning (DL) algorithms for the classification of HIE stages are needed.

### 1.2 Contributions

In this paper, an Anti-Homomorphic Filter (AHF) is proposed to suppress artifacts and noise in the HIE lesion regions of neonatal MR images. This technique is valuable for contrast reduction in overly bright areas of HIE lesion regions. In AHF, the logarithmic domain is reversed, and multiplicative operations are performed to enhance the HIE region in MR images, which are as follows:

1. To enhance the HIE lesion regions in neonatal brain images, the Boston Neonatal Brain Injury Dataset (BONBID) is used for boundary and edge enhancement after the removal of (i) partial volume artifacts, (ii) chemical shift artifacts, and (iii) motion artifacts through the proposed AHF filter.
2. A rough set theory is applied in the neural network to vary the weights of the layers in the proposed RNN-AHF framework for the elimination of voxels caused by adjacent tissues near the HIE lesion region.
3. The HIE lesion regions in brain images are classified according to their stages, Mild HIE, Moderate HIE, and Severe HIE using the proposed optimized training functions: (i) Conjugate Gradient (CG), (ii) Levenberg-Marquardt (LM), and (iii) Reduced Memory Levenberg-Marquardt (RMLM).

### 1.3 Novelty

1. The RNN-AHF framework enhances the HIE lesions in MR images, which are diffuse in nature (*i.e*., multi-focal). HIE is widespread in all tissue regions of the brain in small sizes.
2. Table **1** shows the different algorithms used in the RNN-AHF framework for HIE lesion enhancement.

## 2 RELATED WORKS

Fractional-order derivatives and genetic algorithms are combined to enhance the image through HF [1]. Rayan Al Sobbahi and Joe Tekli [2] developed the Low-Light Homomorphic Filter Network (LLHFNet) for image enhancement. LLHFNet enhances images under both normal-light and low-light conditions. Yin *et al.* [3] enhanced low-light medical images through a combination of algorithms such as HF, gamma correction, and unsharp masking. The above method reduces color distortion. Hu *et al*. [4] used HF and enhanced images through a fusion weight map, which considered the exposure levels and local contrast. The algorithm preserved more details in well-exposed regions and improved edge information. Han *et al.* [5] addressed the limitations of HF on images under complex illumination conditions by developing an exponential HF algorithm. Weighted fusion in the exponential HF algorithm improves image recognition. Yugander *et al.* [6] enhanced noisy MR brain images through a combination of HF and Adaptive Weighted Mean Filtering (AWMF), effectively reducing noise. HF improves contrast by compressing the dynamic range and reducing low-contrast issues in MR images. Pre-processing with AWMF eliminates salt-and-pepper noise before HF. Mustafa *et al.* [7] used HF for image enhancement and increased color accuracy, edge sharpness, brightness, resolution, contrast, and signal-to-noise ratio.

Tao *et al.* [8] analyzed spatial domain image enhancement with HF. HF improves visual quality, brightness, and contrast, which increases performance and accuracy. Agarwal *et al.* [9] developed “Modified Histogram-Based Contrast Enhancement using Homomorphic Filtering” (MH-FIL) for global contrast enhancement. Oh and Hwang [10] combined morphological techniques with HF and improved critical features in images. This technique decomposes the image into morphological sub-bands and applies HF with a structuring element, where optimal gain is precisely used through the differential evolution algorithm. Nnolim and Lee [11] addressed the problem of computational complexity and image enhancement in color images using HF and eliminated frequency-domain transforms and kernel calculations to reduce complexity. Zaheeruddin and Suganthi *et al*. [12] developed a novel image contrast enhancement method using homomorphic decomposition and fuzzy transform. The developed method utilized illumination and reflectance. Homomorphic decomposition extracts illumination from the value layer of the HSV image, while the parametric fuzzy transform enhances the image by updating the membership functions and smoothing the luminance layer. The algorithm enhances images with non-uniform illumination.

### 2.1 Rough Set Theory-based Image Enhancement

Zhang and Dai [13] developed an enhancement algorithm that combines fractional-order differentiation with Rough Set Theory (RST). The integration of the Gaussian mixture model and rough set theory preserves more image details than conventional methods. Zhang X *et al.* [14] used particle swarm optimization (PSO) and RST for image enhancement. The algorithm uses an adaptive fractional differential filter. The resultant images have fine texture details, crisp edges, and preserved information in smooth regions, enhancing image quality.

### 2.2 Neural Network in Image Enhancement

A multimodal information fusion framework combined with neural networks evaluates the therapeutic effects of electroacupuncture moxibustion on cerebral ischemia-reperfusion injury. This method integrates various data modalities, such as clinical parameters and imaging data, to enhance diagnostic accuracy and treatment evaluation. The study underscores the potential of neural networks in processing complex multimodal medical data for therapeutic monitoring [15]. Neural networks, particularly Convolutional Neural Networks (CNNs), are widely used for tasks, such as segmentation, classification, and feature extraction in digitized pathological images. Enhanced image analysis enables accurate disease diagnosis, prognosis prediction, and therapeutic response evaluation. The study also addresses challenges, such as data standardization and annotation strategies, which are crucial for developing robustness [16]. A hybrid approach combining CNNs and ensemble voting classifiers for brain tumor classification using MRI images is proposed [17]. This framework achieved improved diagnostic accuracy by leveraging the feature extraction capabilities of CNNs and the decision-making robustness of ensemble methods. The study highlights the significance of integrating multiple classifiers to enhance performance in medical imaging tasks. A hybrid EfficientNet-DbneAlexNet architecture for brain tumor detection using MRI images is presented. The combination of EfficientNet's scalability and AlexNet's simplicity achieves high precision in tumor detection. The study demonstrates the effectiveness of hybrid architectures in balancing computational efficiency and accuracy for medical image analysis [18]. Deep Convolutional Neural Networks (DCNNs) for brain tumor classification highlight their ability to capture intricate features from MRI scans [19]. This approach has showcased the reliability of DCNNs in identifying tumor types with minimal pre-processing requirements, making them suitable for clinical applications (Nurtay *et al.*, 2025) [19].

### 2.3 Limitations of HF in Medical Images

The disadvantages of Homomorphic filtering (HF) in medical images include noise amplification, loss of detail, parameter sensitivity, limitations in contextual adaptation, computational complexity, and the induction of artifacts. Moreover, these disadvantages persist after the hybridization of algorithms. such as Fuzzy-HF, genetic HF, and MH-FIL.

## 3 METHODOLOGY

Homomorphic filtering (HF) is used in medical imaging to enhance low-contrast MR images. HF improves the visibility of both high-frequency information, such as lesion texture, and low-frequency information, such as illumination or brightness variations in the lesion region. It works by suppressing the reflectance components in MR images. The process involves multiplying the logarithmically transformed image with a homomorphic filter function, which comprises two components: a low-frequency gain and a high-frequency gain. Fig. (**1a**) presents the methodology block diagram, while Fig. (**1b**) illustrates the conceptual framework of the methodology.

### 3.1 Boston Dataset

The Boston Neonatal Brain Injury Dataset for Hypoxic Ischemic Encephalopathy (BONBID-HIE) is used in this study. BONBID-HIE is the first publicly available, comprehensive MRI dataset specifically focused on neonatal brain injury caused by HIE. It includes diffusion MRI scans and expert lesion annotations from 133 term-born neonates diagnosed with HIE, collected retrospectively at Massachusetts General Hospital between 2001 and 2018.

In the proposed RNN-AHF framework, HIE MR images are first processed using the Adaptive Homomorphic Filtering (AHF) method to enhance the HIE-affected regions. The AHF technique effectively enhances widespread, multi-source HIE lesions in MR images, addressing common imaging artifacts such as chemical shifts, motion artifacts, and as chemical shifts, motion. The AHF-filtered image is then processed using a Recurrent Neural Network (RNN), which incorporates Lower Approximation (LA) and Upper Approximation (UA) components. The LA and UA weights are derived using the proposed theorem-based algorithms: (i) LRSM and (ii) NBD RS. The weights obtained from the proposed LRSM and NBD RS algorithms are optimized with PSO, POA, and HO algorithms to remove voxels. The voxel-removed neonatal MR images with HIE lesions are classified into different stages, such as mild, moderate, and severe, using proposed algorithms, such as CG, LM, and RMLM. In this study, the dataset of images is increased using data augmentation methods, such as (i) translation, (ii) scaling, and (iii) flipping. As a result, the image size increases to 5000 images.

### PREAMBLE: ALGORITHMS FOR MULTI-FOCUS HIE LESION

This section explains the global enhancement of HIE lesion MR images and the enhancement of widespread HIE lesions using the following methods: (i) LF_4C, (ii) LF_8C, (iii) GF, (iv) BLF, (v) HF, and (vi) AHF (Proposed).

#### 3.2 Mathematical Analysis of Anti-Homomorphic Filter (AHF) for HIE Region Global Enhancement

In this paper, AHF is used to enhance the HIE region. The high-frequency gain enhances fine details, while the low-frequency gain adjusts illumination levels. By controlling these gains, the filter enables selective emphasis on reflectance details and illumination adjustments without distorting other image regions.

An image 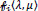 is defined by

**Table:** 

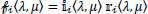	(1)

where 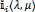 is the illumination component and 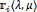 is the reflectance component of objects.

The steps involved in the analysis are as follows:

##### Step 1: Logarithmic Transformation

Take the natural logarithm of the original image to linearize the illumination variations.

**Table:** 

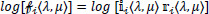	(2)

**Table:** 

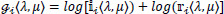	(3)

##### Step 2: Fourier Transformation (FT)

Apply the FT to convert the image into the frequency domain.

**Table:** 

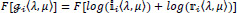	(4)

**Table:** 

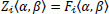	(5)

##### Step 3: Homomorphic Filter Function

Use the HF function 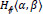 in the frequency domain to generate the filtered version


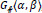
, as described below*:*

**Table:** 

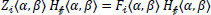	(6)

**Table:** 

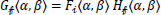	(7)

##### Step 4: Inverse Fourier Transform

Perform an inverse FT to retrieve the filtered image in the spatial domain.

**Table:** 

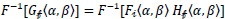	(8)

**Table:** 

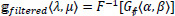	(9)

##### Step 5: Exponential Transformation

Take an exponential transform of the filtered image to revert the linearized image back to its original domain.

**Table:** 

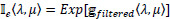	(10)

where 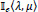 is the enhanced image.

To derive the mathematical equation of AHF, the homomorphic filter function is inverted.

**Table:** 

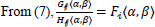	(11)

##### Step 1: Inverse Fourier Transformation

Perform an inverse FT to return the image to the spatial domain.

**Table:** 

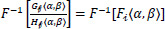	(12)

**Table:** 

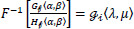	(13)

##### Step 2: Property of Inverse Fourier Transform

**Table:** 

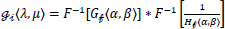	(14)

Calculates the preliminary residual vector

The inverse FT of 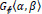 produces the filtered image 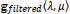, while the inverse FT of 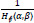 represents the AHF function 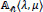. The enhanced image after applying AHF is 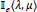.

AHF segregates the illumination (low-frequency) and reflectance (high-frequency) components of the HIE lesion region, allowing for individual processing for enhancement. AHF reverses the spatial information of the HIE region. AHF reduces artifacts in the HIE lesion region in MR neonatal brain images. For partial volume artifact removal, AHF suppresses certain frequency components in an image. Whereas traditional HF enhances both illumination and reflectance variations, AHF eliminates

specific frequency ranges based on the type and level of artifacts, such as (i) partial volume, (ii) chemical shift, and (iii) motion artifact.

##### 3.3 Comparison of the Enhancement Methods on Medical Images

Figs. (**2**-**7**) show the HIE enhancement using the existing and proposed algorithms. Table **2** shows the accuracy of the enhanced medical image using the proposed AHF method.

Table **2** shows a comparison of the proposed AHF method.

##### 3.4 Implication

The proposed AHF method globally enhances the HIE region and removes artifacts, such as partial volume effects, chemical shift, and motion artifacts. Smaller HIE regions require further enhancement, which is achieved through optimized and fine-tuned weights using the RNN.

## 4 PREAMBLE: RNN WEIGHT ESTIMATION AND OPTIMIZED TUNING WEIGHT FOR VOXEL REMOVAL AND HIE BOUNDARY AND EDGES ENHANCEMENT

This section explains voxel removal in HIE images and the enhancement of HIE boundaries and edges using proposed algorithms, such as (i) LRSM, (ii) NBD RS, (iii) POA, (iv) HO, and (v) PSO.

### 4.1 *Weight Estimation of Layers Using RNN for HIE Lesion Boundary and Edge Enhancement* [20]

In RNN, the weights *W_ij_* are represented by lower and upper bounded values to account for uncertainties. A feedforward neural network is applied, achieving stable outputs. The RNN comprises conventional and rough neurons, all of which are fully connected. Each rough neuron consists of two individual neurons: the upper-bound neuron and the lower-bound neuron, as shown in Fig. (**8**). These neurons share information and process different aspects of the input data. The lower bound neuron handles the certain part of the input, generating a lower boundary signal, while the upper bound neuron processes the uncertain part, producing an upper boundary signal based on rough set concepts. This unique lower and upper boundary interpretation is specific to the neural network's learning or training phase, enabling the network to effectively capture and process uncertain data. This approach introduces interval computing in neural networks and presents a new theory for designing neural networks. Fig. (**9**) shows the existing Neural Network weights.

Rough Set Theory (RST) [21] emphasizes approximation and information granulation, partitioning the sample space into information granules using equivalence relations. RST uses Upper Approximation (UA) and Lower Approximation (LA) boundaries to capture uncertainty within information granules, approximating complex knowledge in the sample space. Traditional RST is built upon an indiscernibility relation and an equivalence relation. In this paper, the Indiscernibility Relation (INDR) and Information System (INFS) concepts are proposed as theorems, leading to the development of methods, such as (i) LRSM and (ii) NBD-RS. These theorems are applied in the RNN for enhancing the HIE lesion boundaries and edges using the upper and lower weights.

***Definition 1.*** An INFS is expressed as a quaternion 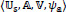, where:


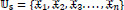
 denotes a universal set containing a finite set of objects [22];


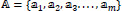
 denotes a finitely defined attribute set;


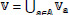
 denotes the set of all attribute values, where 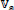 denotes the value range of attribute 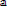;


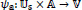
 denotes a mapping function; 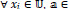

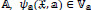
, where 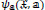 represents an object 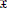
's value on attribute 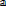.

**Definition 2.** Given an INFS 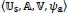, for the set 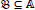, the INDR addressing 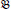 on 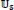 is stated as 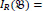

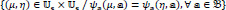
 [23].

The INDR satisfies reflexive, symmetry, and transitive relations. This relation partitions objects into equivalence classes based on their indistinguishability in 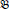. As a fundamental concept in RST, the INDR enables the definition of LA and UA of the set, which are crucial for knowledge approximation in the INFS. We then proceed to define approximations derived from the equivalence class 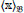.

***Definition 3.*** Let 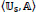 be an INFS with 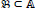, and 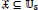. The 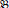-LA and 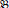-UA are described as follows: 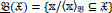 and 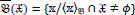.

The 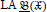 denotes the set that can be classified as belonging to 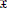 based on 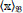 in 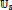. It illustrates the potential 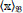 of to approximate the information in 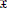 by partitioning the knowledge contained in 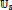. This approximation is known as the 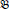-positive region of 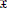 in 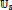, and will be referred to as throughout the paper.

Definition 4. Consider the decision system 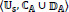 where 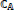 denotes the set of conditional attributes and 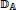 denotes the decision attribute set with 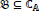 [24]. The positive region of 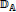, regarding 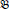, is defined as 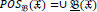. The boundary region is expressed as 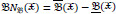.

The magnitude of the positive region serves as an indicator of the classification problem's separability within the given attribute space. An extended positive region enables a more precise representation of the classification problem utilizing the specified attributes. This dependency of 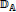 on 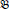 can be quantified as: 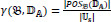, where |·| represents the set cardinality, and 0 ≤ *γ* (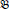, 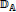) ≤ 1. A larger positive region indicates a more significant reliance of 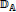 on 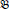. For evaluating attribute sets, this dependency function examines the impact of conditional attributes on classification.

***Definition 5.*** Consider the INFS 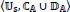. For any, 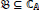 and 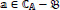, the relative significance of 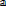 to 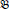 is described as: 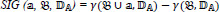.

#### 4.1.1 Accelerating Rough Set Computations by Exploiting the Consistent Local Redundancy of Attributes to Enhance HIE Lesion Boundaries and Edges

Four key theorems for significantly accelerating rough set computations are outlined as follows [25, 26]:

**Theorem 1.** For any 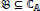 in a decision system 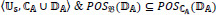.

**Proof.** Stated alternatively, when calculating the positive region for a larger attribute set, computing a subset is unnecessary, optimizing rough set algorithms.

**Theorem 2.** Consider the decision system 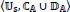, where 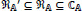 and 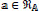 is a specific attribute, with 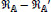 representing a relative reduction of 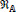. If 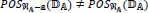 then 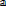 is a non-redundant attribute.

**Proof.** Theorem 2 shows that a non-redundant (core) attribute remains non-redundant in subsets, eliminating the need for re-evaluation. This reduces the computational cost and speeds up the rough set method. Theorems 1 and 2 demonstrate stability in positive regions and non-redundant attributes, enabling the exclusion of redundant attributes and optimizing feature selection.

Theorems 1 and 2 are used for deriving Theorems 3 and 4. Theorems 3 and 4 are used in the LRSM algorithm to estimate the weights of the NN architecture.

***Theorem 3.*** Let 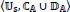 be the decision system with 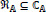 and 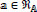. If 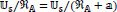, then 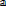 is considered redundant on 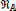.

**Proof.** Theorem 3 reveals that identifying redundant attributes can speed up the rough set process, while strict criteria may limit the overall performance gain. However, even non-redundant attributes often exhibit local redundancy, enabling equivalence class calculations to be skipped. This concept is discussed in the next theorem and elaborated on in the following section.

Following the lead of Theorem 3, definitions of non-active and active regions are:

***Definition 6.*** Consider the decision system 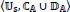 with 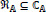 and 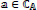 (where 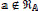) is a specified attribute [27]. The equivalence classes induced by 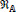 and 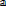 are as follows: 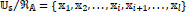

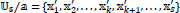
.

For each 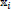 (where i = 1,2,...l), if 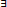 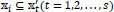, then define: 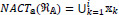 as the non-active region of 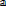 relates 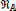, and 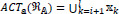 as the active region of attribute 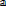 concerning 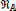


**Theorem 4.** In the decision system 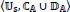, let 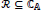 and 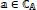 (where 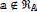). The equivalence classes formed by 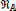 and 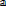 are represented as:
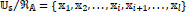

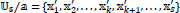
. If 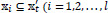 and 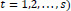, then the non-active region of 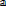 in relation to 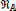 on 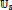 is defined as 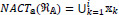 and the active region of 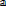 concerning 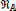 is given by 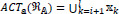.

To assess whether 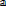 is a non-redundant attribute concerning 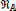, it suffices to concentrate solely on 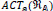.

Theorem 4 reveals that the non-active region of an attribute is irrelevant to further computations, regardless of redundancy. This insight leads to significant performance optimization, as calculations are streamlined and unnecessary regions are omitted, enhancing the boundary and edges of HIE.

**Definition 7.** Given INFS 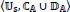, and 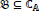 (where 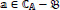), the relative significance of 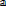 relating 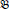 defines, *RSIG*(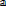,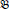,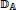) = 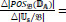

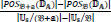


Here |.| denotes the cardinality of the set, and ∆ indicates the difference operator, reflecting the change in the number of elements. A higher number of positive region points can simplify decision boundaries and create more equivalence classes, which leads to overfitting. To address this problem, the attribute importance in rough sets is evaluated using relative importance, which considers both the positive region and equivalence classes.

Utilizing the foundational theorems on attribute redundancy stability and the relative importance model, the Local Redundancy Rough Set Stability Measure (LRSM) framework enhances traditional rough set methods. This proposed framework consists of five essential stages, as illustrated in Fig. (**10**).

**Fig. (10):**
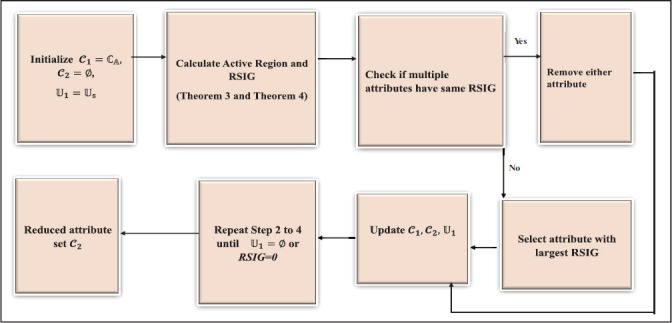
A Flow chart of the LRSM framework.

As illustrated in the flowchart (Fig. **10**), Algorithm 1 outlines the steps of the new rough set approach. This method incorporates a model that assesses stability and attribute importance by considering local redundancy and improves the edges and boundary of HIE lesions.

### Proposed: Weight Estimation Using LRSM Algorithm (Active Region) for NN

**Input:** Decision system 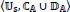


**Output:** Reduced set *C*_2_

**Step 1:** Start up *C*_1_ = 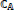 and *C*_2_ = 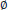.

**Step 2:** For each attribute 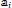 in *C*_1_:

a) Figure out the significance of 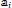 using Theorem 4

b) If the significance is 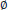, remove 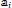 from *C*_1_ (Theorem 3)

**Step 3:** Pick the attribute 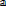 that maximizes the relative importance (max RSIG) from the set *C*_1_

**Step 4:** If 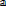 has a positive relative importance (greater than 0):

a) Include 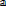 to *C*_2_

b) Remove 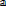 from *C*_1_

c) Update 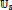 by removing the positive region under 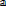 (Theorem 1)

**Step 5:** If 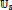 is not empty, repeat steps 2-4. Otherwise, return *C*_2_.

To elucidate the LRSM algorithm, assume that the universal set contains eight elements, 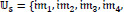

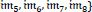
. Let the conditional attribute set be 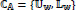 and 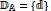 be the decision attribute set. This forms a decision system 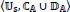, as illustrated in Table **3**.

From Table **3**, the following outcomes can be determined.


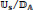
 = {{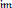
_1_,
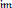
_3_,
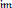
_6_,
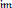
_8_},{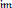
_2_,
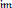
_4_,
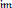
_5_,
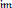
_7_}},


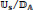
 = {{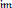
_1_},{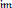
_2_,
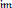
_3_},{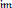
_4_},{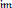
_5_},{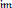
_6_,
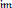
_8_},{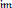
_7_}},


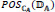
 = {{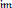
_1_,
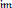
_4_,
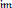
_5_,
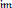
_6_,
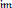
_7_,
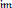
_8_}.


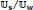
 = {{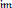
_1_,
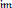
_2_,
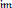
_3_},{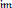
_4_},{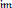
_5_},{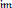
_6_,
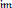
_8_},
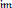
_7_}},


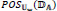
 = {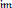
_4_,
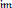
_5_,
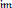
_6_,
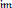
_7_,
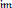
_8_}.


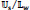
 = {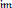
_1_},{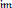
_2_,
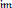
_3_,
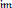
_4_},{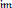
_5_},{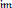
_6_,
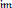
_8_},{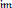
_7_}}


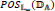
 = {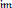
_1_,
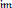
_5_,
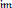
_6_,
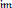
_7_,
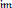
_8_}.

According to Definition 6,


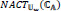
 = {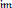
_1_,
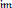
_5_
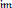
_6_
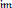
_7_
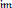
_8_}


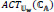
 = {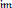
_2_,
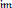
_3_,
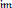
_4_}.


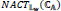
 = {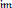
_4_,
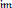
_5_,
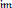
_6_,
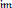
_7_,
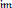
_8_},


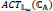
 = {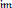
_1_,
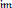
_2_,
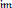
_3_}.

*RSIG*(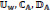) = 1, *RSIG*(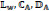) = 1.

In this case, both 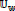, 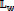 have the same RSIG of 1. So, we can remove anyone attribute and 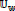 is non-redundant attribute.

#### 4.1.2 *Neighbourhood Rough Set (NBD-RS) for Weight Estimation of NN* [28]

The proposed NBD-RS is based on classical rough set theory and addresses uncertainty in data, especially in cases involving continuous or mixed data types. NBD-RS establishes a neighbourhood around each object determined by a defined radius. This approach enables a more adaptable depiction of indiscernibility among objects.

**Definition 8** [29]. A metric *∆*_*Ω*_ on a set *Ω* is a function *∆*_*Ω*_: *Ω* × *Ω* -> *R* such that 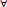 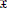
_1_, 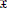
_2_ ϵ *Ω*:

● Non-negativity: 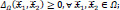


● Identity: 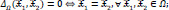


● Symmetry: 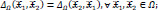


● Triangle inequality: 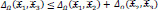


**Definition 9.** Let 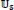 = {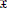
_1_,
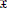
_2_,
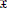
_3_,...,
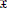
_n_} be the universal set finitely [28]. 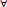 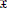
_*i*_ϵ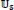, *δ* ≥ 0, the *δ*-neighborhood of 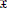
_*i*_ is defined as *δ*(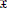
_*i*_) = {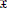
|
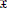
ϵ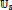,∆*_Ω_*(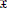,
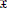
_*i*_) ≤ *δ*.

***Definition 10.*** Let 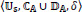 be the neighborhood decision system (NBD-DS) [28]. For the equivalence classes *X*_1_,*X*_2_,*X*_3_....,*X*_L_ and 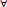,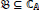, the LA and the UA of 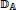 in relation to 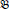 are respectively described as: 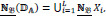

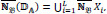
 where 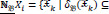

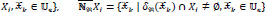
. The positive region and the boundary region are also defined as 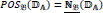

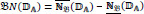
.

***Definition 11.*** Consider the NBD-DS 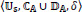 with 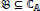. The conditional information entropy (CIE) associated with 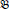 is defined as: 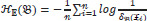


***Definition 12.*** Suppose that 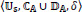 is the NBD-DS [28]. The CIE of 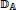 on 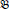 is stated as follows:


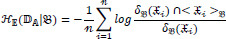


**Definition 13.** Let the NBD-DS be 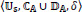. The following definition indicates the attribute importance of 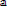 regarding 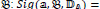

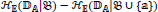
.

### Proposed: Neighbourhood Rough Set (NBD RS) Based on Attribute Importance Algorithm for Weight Estimation of Layers

**Input:** Decision system 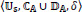 radius *δ*.

**Output:** The required resultant set is 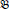


**Step 1:** Initialize 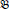 = 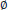.

**Step 2:** Compute the CIE 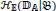 for the entire NBD-DS.

**Step 3:** Repeat:

1) For each 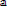
*_i_*,(*i* = 1,2,3...n), find the CIE 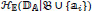;

2) Determine the attribute importance 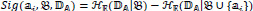


3) Pick the most important attribute 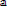
*_i_*, where 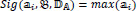


4) If 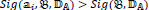, add 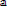
*_i_* to the resultant set 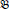 then move to Step 3.

**Step 4:** Provide the resultant outcome.

### 4.2 Tuning of RNN Weight Estimation

#### 4.2.1 Pelican Optimization Algorithm (POA)

The Pelican Optimization Algorithm (POA) simulates the hunting behaviors of pelicans. In this algorithm, virtual pelicans represent search agents, exploring the solution space and locating food sources (optimal solutions). The process involves initializing a population of pelicans, evaluating their fitness based on an objective function, and iteratively updating their positions through exploration and exploitation phases. POA effectively balances exploration and exploitation, making it suitable for solving various optimization problems.

#### 4.2.2 Hippopotamus Optimization (HO) Algorithm

Hippopotamus Optimization (HO) algorithm mimics the behaviors of hippopotamuses. HO uses a three-phase model, incorporating position updates, defensive strategies against predators, and evasion methods. The algorithm utilizes a population of hippopotamuses as candidate solutions and updates their positions based on performance evaluations.

#### 4.2.3 Particle Swarm Optimization (PSO) Algorithm

Particle Swarm Optimization (PSO) simulates the social behavior of birds or fish. It employs a swarm of particles, each representing a potential solution, that moves through the solution space. Each particle adjusts its position based on its personal best and the global best solutions found by the swarm. The movement is influenced by velocity, allowing particles to explore new areas while refining their search around promising solutions. PSO effectively balances exploration and exploitation, making it suitable for a wide range of optimization problems across various domains.

##### 4.2.3.1 Implications

- Among the proposed weight estimation algorithms, LRSM performs better due to high stability and enhancement of the edges and boundary of the smaller size HIE region.
- The estimated weight needs to be tuned to remove the voxels, and the proposed PSO algorithm performs better due to the exploitation phase.

## 5 PREAMBLE: HIE STAGE CLASSIFICATION

This section explains that the enhanced HIE images are classified using proposed algorithms such as (i) CG, (ii) LM (iii) RMLM.

### 5.1 Optimized Training Function

#### 5.1.1 Proposed: Conjugate Gradient (CG) Algorithm for HIE Lesion Stage Classification

The CG algorithm solves linear equations and large, sparse, symmetric, non-negative definite matrices. The CG algorithm excels in converging with a minimal number of iterations compared to other iterative methods.

The concept of the CG algorithm is to solve the system of linear equations 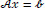, where *A* is a symmetric non-negative definite matrix with the sequence of vectors {*x*_0_,*x*_1_,*x*_2_.......*x*_*n*_}.

The CG algorithm steps are as follows:

1. Boot up with the solution, *x*_0_.

2. Calculate the preliminary residual vector

**Table:** 

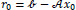	(16)

3. Set the first search direction

**Table:** 

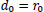	(17)

4. For each iteration *k*,(*k* = 0,1,2.....), do the following until convergence:

a. Calculate the step size *α_k_*:

**Table:** 

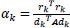	(18)

b. Update the solution:

**Table:** 

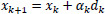	(19)

c. Calculate the new residual vector:

**Table:** 

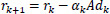	(20)

d. Calculate the new search direction:

**Table:** 

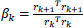	(21)

**Table:** 

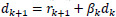	(22)

5. Repeat the iterations until the residual is small enough or a predefined number of iterations is reached.

The condition number of the matrix *A* plays a significant role in determining the convergence speed of the CG algorithm.

#### 5.1.2 Proposed: Levenberg-Marquardt (LM) Algorithm for HIE Lesion Stage Classification

The LM algorithm optimizes non-linear least squares problems. The algorithm combines the Gauss-Newton method and the gradient descent method. It adapts the step size based on the local curvature of the objective function to converge and maintain stability. The steps of the LM algorithm are explained below:

A non-linear least squares problem involves data points (*x_i_*, *y_i_*) and a model function 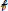
(*x_i_*,*β*), where *β* is a vector of parameters for estimation. The objective is to find *β* that minimizes the sum of squared errors:

**Table:** 

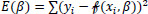	(23)

Step 1: Linearize the model function

The LM algorithm linearizes the model function around an initial estimate of the parameter vector *β*.

Linearized model:

**Table:** 

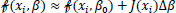	(24)

Where,

*β*_0 _: Initial estimate of the parameter vector.

*J*(*x_i_*): The Jacobian matrix of 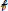
(*x_i_*,*β*) with respect to *β* evaluated at *β*_0 _.

∆β : The change in the parameter vector β.

Step 2: Update the parameter vector

The update equation is based on minimizing the squared errors using the linearized model:

**Table:** 

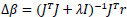	(25)

Where,

*J^T^*: Transpose of the Jacobian matrix*.*

*r*: Residual vector,

**Table:** 

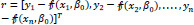	(26)

*λ*: The damping factor (or regularization parameter) that manages the compromise between the gradient descent step (when *λ* is large) and the Gauss-Newton step (when *λ* is small).

*I*: The identity matrix.

Step 3: Update the parameter vector *β*,

**Table:** 

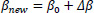	(27)

Step 4: Update the damping factor *λ*

The damping factor *λ* is updated based on the success of each iteration. If the new parameter vector reduces the error, the damping factor is decreased to take larger steps towards convergence.

If the error decreases:

**Table:** 

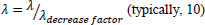	(28)

If the error increases:

**Table:** 

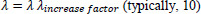	(29)

Step 5: Repeat steps 1-4 until convergence.

The iterations continue until the parameter vector *β* converges to the optimal solution or a predefined termination condition is fulfilled (*e.g.*, a maximum iteration count or a minimal tolerance value).

#### 5.1.3 Proposed: Reduced Memory Levenberg-Marquardt (RMLM) Algorithm for HIE Lesion Stage Classification

The RMLM algorithm is a memory-efficient variant of the LM algorithm, addressing the challenges of large-scale optimization problems where memory resources are limited.

The RMLM algorithm improves the approximation of the Hessian matrix and stores a limited number of vectors (known as “memory”) to represent the history of the optimization process. This algorithm uses historical information from previous iterations without storing the full Jacobian or the complete history of the optimization process.

The main steps of the Reduced Memory Levenberg-Marquardt algorithm are as follows:

1. Set up an initial parameter, *p*_0 _.
2. Evaluate the objective function and calculate the gradient vector based on the current parameter values.
3. Initialize the memory with a set of vectors {*s*_1_,*y*_1_,....,*s_m_*,*y_m_*}, where `m' is the number of memory vectors and is typically much smaller than the number of parameters.
4. At each iteration, the algorithm performs the following steps:
  - Computes the search direction using the L-BFGS update, which approximates the inverse Hessian matrix.
  - Updates the parameter vector using the search direction and a suitable step size.
  - Renews the memory by adding a new pair of vectors (*S_k_*,*y_k_*), where *S_k_* is the change in parameters and *y_k_* changes the gradient between the current and previous iterations.
5. After updating the parameter vector, recalculate the objective function and check for convergence. If the convergence criteria are not met, repeat steps 2 to 4 until convergence is achieved.

The RMLM algorithm is used for problems of lean parametrization compared to the data points since the L-BFGS approximation may not be as accurate in high-dimensional parameter spaces.

### 5.2 Implications

Globally and locally enhanced smaller-size HIE regions need to be classified for their stage, and the proposed RMLM algorithm performs better due to the Hessian matrix.

## 6 RESULTS

Table **4** summarizes the statistical information of the datasets used in the study, providing details on the total number of samples, training and testing splits, and the image sizes for each dataset.

Table **5** displays the accuracy metrics for the BONBID-HIE dataset using various filters and optimization algorithms. The advantages of AHF stem from its approach to dynamic range manipulation in image processing, particularly in contexts where the imaging system or display media inherently apply nonlinear transformations to light intensity values. Unlike HF, which compresses dynamic range by applying a logarithmic transform before filtering, AHF first expands the dynamic range to recover the true light intensity values, applies linear filtering, and then compresses the dynamic range back for display.

Table **6** summarizes the duration of processing time (in minutes and seconds) for the PCam dataset using multiple filters under various optimization algorithms. The rows represent different filters, such as AD, LF_8C, BM3D, Gaussian, Homomorphic, Mean, TV, Wavelet, and Anti-Homomorphic, while the columns detail the elapsed times for three methods. After filtering, the dynamic range is compressed back to the display domain, which often has an anti-logarithmic or expansive response. This ensures that the final image is visually consistent with human perception and display device characteristics.

Table **7** presents a comparison of models from the literature on relevant datasets, including PCam, RFMID, and BONBID-HIE. RST is particularly effective in addressing the inherent uncertainty and imprecision in MR images caused by noise, artifacts, or partial volume effects. It uses LA and UA to classify data into definite and possible regions, enabling robust segmentation and enhancement even in challenging scenarios like bias field correction and tissue overlap.

Table **8** provides a summary of related works on neonatal brain lesion analysis utilizing machine learning methods. It includes references, the type of analysis (*e.g.*, segmentation or detection), approaches used, features extracted, performance metrics, datasets, contributions, and observations.

Table **9** summarizes various DL-based methodologies for neonatal brain lesion analysis, highlighting approaches, features, performance metrics, datasets, contributions, and observations. It includes segmentation techniques, such as PAU-Net, HTTU-Net, and Triple Intersecting U-Net, which utilize Convolutional Neural Networks (CNN) to increase the performance of datasets, such as BRATS and Brain Web. The proposed methods emphasize innovations, such as hybrid loss functions, multi-level feature extraction, and novel architectural designs to enhance accuracy and efficiency.

Table **10** provides a summary of hybrid methods used for brain lesion analysis, combining Machine Learning (ML) and Deep Learning (DL) techniques. It includes approaches such as PGGAN, DNN, K-means, and Bayesian fuzzy clustering, applied to tasks such as segmentation, classification, and detection across datasets like BRATS, AANLIB, and MRI scans. These methods leverage existing techniques such as discrete wavelet transform (DWT), principal component analysis (PCA), and generative adversarial networks (GANs) to enhance accuracy, sensitivity, and specificity.

AHF is designed to compensate for nonlinear intensity transformations in imaging systems by expanding the dynamic range before filtering and compressing it afterward. This process enables more effective linear filtering on true intensity values. While AHF focuses on dynamic range manipulation, rough set-based filters emphasize uncertainty modeling and adaptive smoothing. Combining these two approaches could theoretically create a robust MRI enhancement framework. AHF would correct nonlinear intensity distortions and enhance contrast, while rough set-based filters would adaptively smooth and preserve edges by classifying pixel regions based on uncertainty and rough approximations. Such a hybrid approach would leverage AHF’s dynamic range correction and rough sets’ noise-handling and edge-preservation capabilities, potentially improving MRI image clarity and diagnostic utility.

Table **11** presents performance metrics, such as specificity, sensitivity, and accuracy achieved using various methods. Mathematical morphology, which focuses on shape-based image processing, shares conceptual similarities with rough set approaches in handling spatial structures and noise. Morphological filters remove noise while preserving object shapes, similar to rough set filters that use approximations to retain important image features. These methods complement AHF by addressing spatial noise and structural preservation. Rough set-based filters enhance MRI image quality by adaptively smoothing based on uncertainty modeling, while AHF improves images through dynamic range expansion and compression, allowing more effective linear filtering. A hybrid approach combining AHF and rough set filtering could leverage the strengths of both: dynamic range correction alongside adaptive noise reduction and edge preservation. Additionally, morphological methods, which are related to rough sets, support noise removal without altering object shapes.

## 7 DISCUSSION

Image enhancement and classification of HIE lesions in neonatal brain images are challenging due to unclear boundaries and edges, which result from chemical shifts, partial volume artifacts, motion artifacts, and voxel reflections from adjacent tissues. Existing algorithms often produce inaccurate tissue representations due to chemical shift artifacts, where bright and dark bands are unclear, leading to misinterpretations of anatomical structures because of blurring and distortion. To address this issue, an efficient filter for tissue classification and Deep Learning (DL) algorithms for stage classification are required. Therefore, the RNN-AHF approach is proposed. Anti-Homomorphic filtering enhances medical images by manipulating their frequency content while preserving the overall structure. AHF effectively removes both low- and high-frequency noise, making it particularly suitable for medical image analysis. It segregates the illumination and reflectance of tissue regions, reverses the spatial information of these regions, and reduces artifacts in the HIE lesion area.

AHF suppresses specific frequency components, enhancing both illumination and reflectance variations, while eliminating or attenuating the frequency ranges responsible for partial volume, chemical shift, and motion artifacts. The RNN-AHF framework reduces the pixel dimensionality of the feature space, eliminating unnecessary pixels and preserving the essential ones for enhancement. The RNN efficiently learns and identifies pixel patterns, enabling adaptive enhancement by adjusting weights within the neural network.

In the RNN-AHF framework, the HIE region is first enhanced using AHF. Then, RNN is employed to obtain upper and lower weights using two proposed algorithms: (1) LRSM and (2) NBD-RS. These weights are optimized using additional algorithms, including (1) POA, (2) HO, and (3) PSO, which are then input into the RNN architecture. For feature extraction from the image, the training functions used are (1) RMLM, (2) CG, and (3) LM. The combination of these proposed algorithms leads to a more accurate classification of HIE levels, with the AHF-LRSM-POA-RMLM approach achieving a high accuracy of approximately 93.5%.

## CONCLUSION

In this study, we addressed the fusion of AHF with RNN and provided a highly effective image enhancement of the HIE region. By addressing both noise suppression and redundancy reduction, this approach enhances key visual features, leading to improved image quality and clarity. The RNN further refines the enhancement process, making it adaptable to various image types and noise levels. The demonstrated improvements in visual fidelity and computational efficiency highlight the potential of this RNN-AHF framework for HIE image enhancement. The RNN-AHF framework enhances the diffuse nature of HIE lesions. HIE is widespread in all tissue regions of the brain in small sizes. The spread of HIE lesions in multi-spread regions is enhanced with the RNN-AHF framework. In this framework, AHF enhances the global HIE regions in MR images and removes HIE lesion artifacts such as chemical shifts, motion artifacts, and partial volume artifacts. LA and UA weights are obtained with proposed algorithms, such as (i) LRSM and (ii) NBD-RS. The obtained weights from the proposed LRSM and NBD-RS algorithms are optimized with PSO, POA, and HO algorithms to remove voxels in HIE lesion regions. RMLM, CG, and LM algorithms classify the HIE stages. Furthermore, the dataset of images can be increased with GAN, and with the help of GAN algorithms, the size of the lesions can be further reduced to very small sizes, allowing HIE to be detected at earlier stages.

## Figures and Tables

**Fig. (1a):**
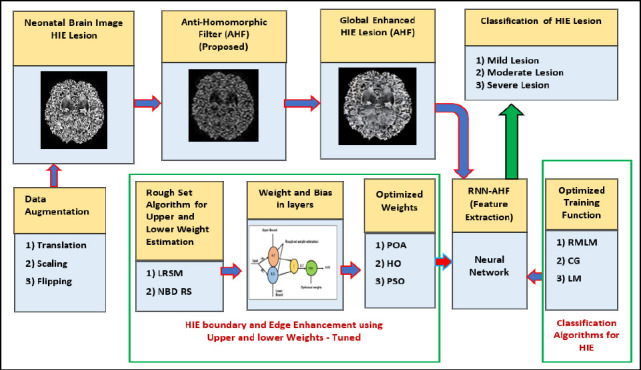
Methodology of RNN-AHF framework.

**Fig. (1b):**
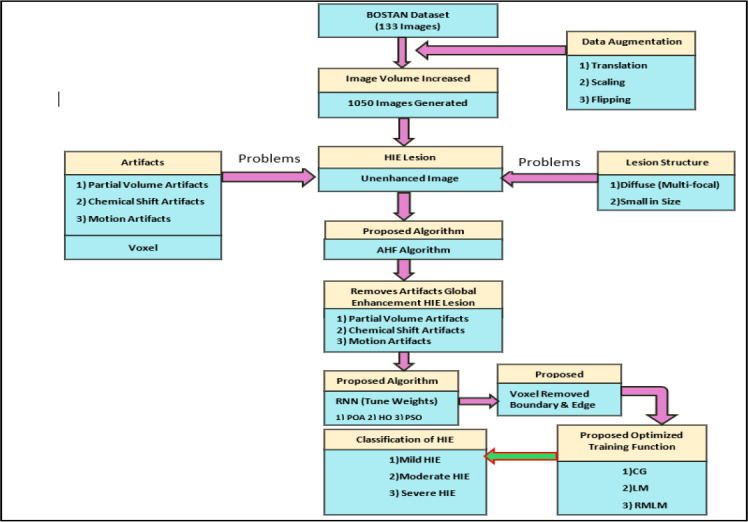
Conceptual diagram of proposed methodology.

**Fig. (2):**
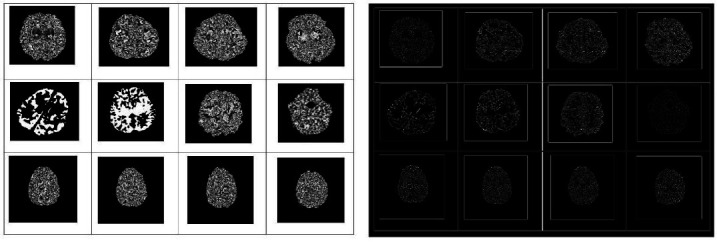
Enhanced image using existing Laplacian 4C Filter (LF_4C) method.

**Fig. (3):**
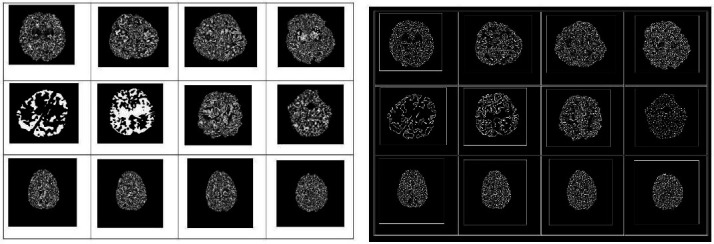
Enhanced image using existing Laplacian 8C Filter (LF_8C) method.

**Fig. (4):**
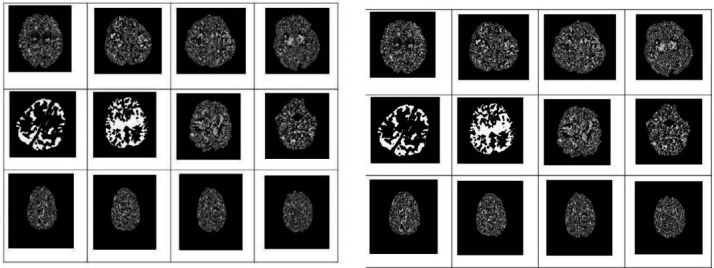
Enhanced image using existing Gaussian Filter (GF) method.

**Fig. (5):**
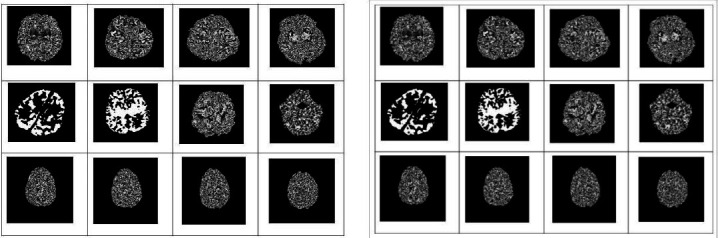
Enhanced image using existing Bilateral Filter (BLF) method.

**Fig. (6):**
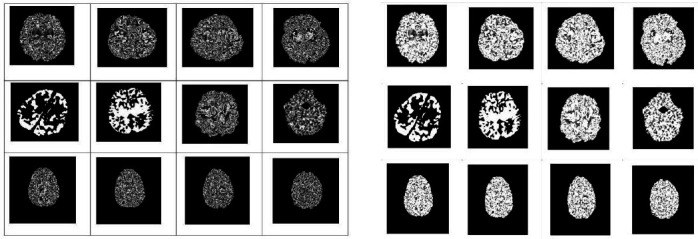
Enhanced image using existing Homomorphic Filter (HF) method.

**Fig. (7):**
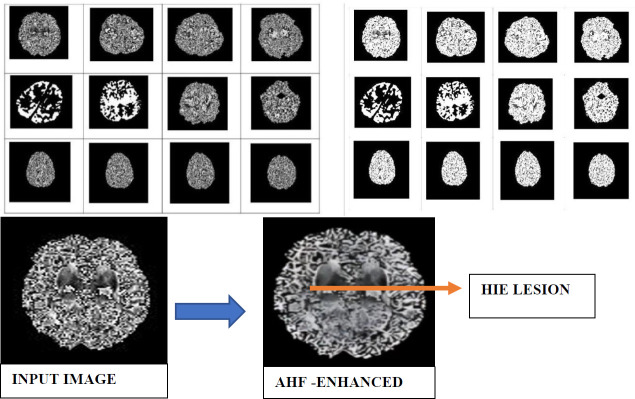
Enhanced image using proposed Anti-Homomorphic Filter (AHF) method.

**Fig. (8):**
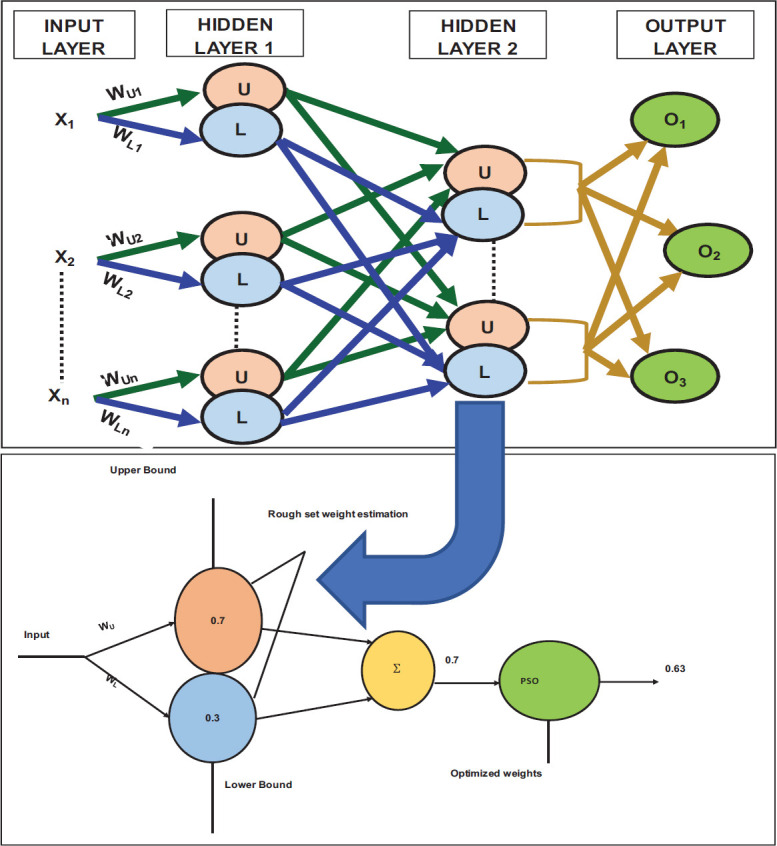
Rough neural network with rough neurons (Proposed).

**Fig. (9):**
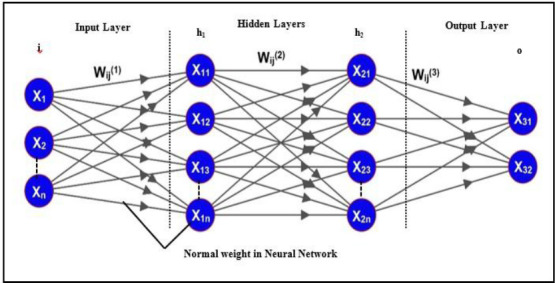
Neural network (Existing).

**Table 1:** RNN-AHF framework.

**RNN-AHF Framework**				
**Parameter**	**Filter (Proposed)**	**Weight Estimates**	**Tuning of Weight**	**Feature Extraction**
BONBID-HIE (DATASET)	AHF	RNN Algorithm (Proposed)	Tuning (Proposed)	Training function (Proposed)
		LRSM	PSO	CG
		NBD-RS	HO	LM
			POA	RMLM
Novelty	Small-size HIE lesions widespread in the multi-focus form are enhanced, and the chemical shift, motion artifacts, and partial volume artifacts are removed.	Edges and Boundaries of HIE Enhancement	Voxels removed	Classification of HIE stages

**Table 2:** Accuracy of enhanced medical image using proposed AHF method.

**Parameters**	**LF_4C** **(Existing)**	**LF_8C (Existing)**	**GF (Existing)**	**BLF (Existing)**	**HF (Existing)**	**AHF (Proposed)**
Accuracy	0.6671	0.6931	0.9801	0.9762	0.9348	0.9350
False Negative (FN)	576	3914	12231	14666	61223	61164
False Positive (FP)	313642	285800	6546	7817	324	232
FP Rate	0.4851	0.4644	0.0200	0.0239	0.0012	0.000851
F Value	0.6539	0.6915	0.9847	0.9817	0.9520	0.9521
Jaccard Index	0.4858	0.5284	0.9698	0.9640	0.9084	0.9086
Mathews Correlation Coefficient (MCC)	0.4985	0.5219	0.9564	0.9478	0.8605	0.8609
Precision	0.4862	0.5318	0.9893	0.9872	0.9995	0.9996
Recall	0.9981	0.9881	0.9801	0.9762	0.9088	0.9089
True Negative (TN)	332974	329636	321319	318884	272327	272386
True Positive (TP)	296808	324650	603904	602633	610126	610218
TP Rate	0.9981	0.9881	0.9801	0.9762	0.9088	0.9089

**Table 3:** Decision system 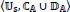.

**Images**	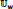	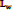	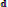
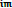 _**1**_	0.7	0.7	1
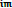 _**2**_	0.7	0.3	0
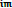 _**3**_	0.7	0.3	1
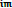 _**4**_	0.3	0.3	0
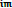 _**5**_	0.2	0.2	0
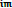 _**6**_	0.5	0.5	1
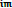 _**7**_	0.4	0.4	0
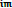 _**8**_	0.5	0.5	1

**Table 4:** Statistical overview of the datasets employed in this research.

**Dataset**	**Total Data**	**Train**	**Test**	**Image size**
PCam	19,520	15,616	3904	50x50 (RGB)
RFMID	3,200	2560	640	320x256 (RGB)
BONBID-HIE (Proposed)	133	89	44	256x256 (Gray)

**Table 5:** Accuracy results for the bonbid-hie dataset across multiple filters.

**Data set**	**Filters**	**Local Redundancy Rough Set Stability Measure+Pelican Optimization Algorithm+Reduced Memory Levenberg-Marquardt Algorithm (LPR)**	**Neighborhood Rough Set+Hippopotamus Optimization Algorithm+Conjugate Gradient Algorithm (NHC)**	**Local Redundancy Rough Set Stability Measure+Partial Swarm Optimization Algorithm+Levenberg-Marquardt Algorithm (LPL)**
BONBID-HIE (Proposed)	AD	0.739	0.827	0.673
	LF_8C	0.693	0.811	0.658
	BM3D	0.741	0.819	0.686
	Homomorphic	0.934	0.829	0.709
	Mean	0.740	0.826	0.674
	TV	0.735	0.807	0.643
	Wavelet	0.743	0.821	0.617
	Anti-Homomorphic (Proposed)	0.935	0.836	0.908

**Table 6:** The elapsed time (Minutes: Seconds) performance of the BONBID-HIE dataset under various filtering systems.

**Data set**	**Filters**	**Local Redundancy Rough Set Stability Measure+Pelican Optimization Algorithm+Reduced Memory Levenberg-Marquardt Algorithm (LPR)**	**Neighbourhood Rough Set+Hippopotamus Optimization Algorithm+Conjugate Gradient Algorithm (NHC)**	**Local Redundancy Rough Set Stability Measure+Partial Swarm Optimization Algorithm+Levenberg-Marquardt Algorithm (LPL)**
BONBID-HIE (Proposed)	AD	0:49	1:51	3:31
	LF_8C	0:58	1:49	3:45
	BM3D	0:52	1:44	3:38
	Homomorphic	0:38	1:35	3:11
	Mean	0:42	1:38	3:27
	TV	0:43	1:48	3:36
	Wavelet	0:39	1:50	3:31
	Anti-Homomorphic (Proposed)	0:36	1:32	3:09

**Table 7:** Comparative analysis with existing literature studies on analogous datasets.

**Dataset**	**Source**	**Model**	**Train-Test Split (%)**	**Accuracy**	**Precision**	**Recall**	**F1-score**	**AUC**
PCam	Struski *et al.* (2024) [30]	ImpoMIL	85-15	0.876	-	-	-	0.857
	Furkan Atlan *et al.* (2024) [31]	EfficientNetB0 + SVM (BM3D filter)	80-20	0.805	0.805	0.805	0.805	0.876
	Stegmuller *et al.* (2023) [32]	ScoreNet	5-fold cross validation	0.746	-	-	-	0.736
	Sun *et al.* (2023) [33]	TGMIL	5-fold cross validation	0.879	0.894	-	0.875	0.898
RFMID	Choi *et al.* (2024) [34]	EfficientNetB0+StyleGAN2	80-20	-	-	0.961	-	0.951
	Furkan Atlan *et al.* (2024) [31]	ResNet50v2 þ SVM (Bilateral filter)	80-20	0.903	0.903	0.903	0.903	0.941
	Al-sebaay *et al.* (2023) [35]	Inceptionv3	80-20	0.970	0.866	0.680	0.762	0.936
	Sengar *et al.* (2023) [36]	EyeDeepNet	78-22	0.821	0.768	0.761	0.761	-
BONBID-HIE (Proposed)	RNN-AHF	Local Redundancy Rough Set Stability Measure+Pelican Optimization Algorithm+Reduced Memory Levenberg-Marquardt Algorithm (LPR)	89-44	0.935	0.999	0.908	0.952	0.960

**Table 8:** Compilation of research on brain lesion analysis with ml methods.

**Reference**	**Type**	**Approach**	**Features**	**Performance**	**Dataset**	**Observations**
Anantha rajan *et al.* (2021) [37]	Tumor stage detection	Weighted FCA	LBP	Accuracy, 91.84	MICCAI	The utilized feature lists lacked clarity
		DAE	GLCM	Sensitivity, 89.9		Clear information about the results and scores were not available
		BMOA	High-order pixel features	Specificity, 93.37		Dataset information was vague
		Random forest		F-measure, ~0.9		
Kumar *et al.* (2021) [38]	Detection	AKNN	GLCM	MCC,0.9	BRATS	Improved upon the results of existing models
	Segmentation	OP-FCM		F1-Score, 0.9	Internet	Offered an intuitive interface for performance evaluation
		BCSO		Accuracy, 91.9		Achieved performance similar to or better than DL models
				Sensitivity, ~90.2		Failed to provide a proper description of the dataset used
				Specificity, ~ 98		
Proposed RNN-AHF	Segmentation	LPR	RNN-AHF	Accuracy, 93.5	BONBID-HIE	Integration of AHF with LRSM suppresses noise and reduces redundancy to improve clarity of images
		NHC		Sensitivity, 90.89		RNN refines the enhancement making it adaptable to various images
		LPL		Specificity, 99.91		High Computational efficiency

**Table 9:** Overview of relevant literature on brain lesion analysis using DL methods.

**Reference**	**Type**	**Approach**	**Features**	**Performance**	**Dataset**
Aboelenein *et al*. (2020) [39]	Segmentation	HTTU-Net	CNN	DCC, 0.75-0.87	BRATS 2018
Zhang *et al*. (2021) [40]	Segmentation	Triple intersecting U-Net	CNN	DCC, 0.63-0.85 Mean rank, 1.25	BRATS 2015
				DCC, 0.99 PA, 0.99 Mean rank, 2.67	Brain Web
Shariff *et al*. (2020) [41]	Feature selection	Contrast stretching	SFTA	Accuracy, 90.45-92.8	BRATS 2013
	Segmentation	Salient-based segmentation	LBP deep CNN	DCC, 95.42	BRATS 2014
	Modality Classification	PSO	DRLBP		BRATS 2017
		Inception V3			BRATS 2018
Sun *et al*. (2021) [42]	Segmentation	Multi-pathway	CNN	DCC, 0.76-0.89	BRATS 2018
		FCNN			BRATS 2019
RNN-AHF (Proposed)	Segmentation	LPR	RNN-AHF	Accuracy, 93.5	BONBID-HIE
		NHC		Sensitivity, 90.89	
		LPL		Specificity, 99.91	

**Table 10:** Summary of prior studies on brain lesion analysis using hybrid methods.

**Reference**	**Type**	**Approach**	**Features**	**Performance**	**Dataset**	**Observations**
Han *et al.* (2020) [43]	Synthetic brain	PGGAN	CNN	Accuracy, 91.08	BRATS 2016 (T1c)	Provided a reliable Solution to the problem of small datasets through the generation of synthetic data
	MRI generation			Sensitivity, 86.6		
	Data augmentation			Specificity, 97.6		
	Tumor detection					
Khan *et al.* (2021) [44]	Classification	K-means VGG19	-	Accuracy, 90.03-92.06	BRATS 2015	Categorized tumors as benign/ malignant
						Implemented transfer learning approach
						Assessed performance using data augmentation and without it
						Clustering parameters were not fully specified
Raja *et al.* (2020) [45]	Segmentation	DAE	Information theoretic	Accuracy, 92.35	BRATS 2015	Achieved superior performance compared to 17 existing models across conventional, ML, DL, and hybrid methods
	Classification	BFC	WPTE			The list of applied features was unclear
			ST			
Preethi *et al.* (2021) [46]	Detection	DWT	GLCM	Accuracy, 92.9	MRI	Advanced a pioneering fusion rule
	Segmentation	Novel Fusion		Sensitivity, 89	PET (Internet)	Showed better outcomes of similar methods
		SMO		Specificity, 93		The dataset details were incomplete
		ODNN				Provided the theoretical framework, but the experimentation details were not clearly outlined
		Weighted K-means				
RNN-AHF (Proposed)	Segmentation	LPR	RNN-AHF	Accuracy, 93.5	BONBID-HIE	Integration of AHF with LRSM suppresses noise and reduces redundancy to improve clarity of images
		NHC		Sensitivity, 90.89		RNN refines the enhancement making it adaptable to various images
		LPL		Specificity, 99.91		high computational efficiency

**Table 11:** Dataset used in brain lesion analysis.

**Dataset**	**Specificity**	**Sensitivity**	**Accuracy**	**Method**
BRATS 2015	98.7	0.902	0.92	Region-growing
				SVM
				GOA
BRATS 2015 BRATS 2019	DCC, 0.69-0.89	VD, 3.91-6.13	DCC, 86%	Binary decision Tree
				Random forest
				WHT
				SLIC saliency map
				Spectral clustering (K-means) Connected component
BRATS	98	90.07	91.9	AKNN
				OP-FCM
				BCSO
BRATS 2017	DCC, 0.6-0.92	0.64-0.87	PPV, 0.92-0.93	PAU-Net
BRATS 2018				
BRATS 2013	DCC, 95.42		90.5-92.3	Contrast stretching
BRATS 2014				Segmentation based on saliency
BRATS 2017				PSO
BRATS 2018				Inception V3
BRATS 2018	DCC, 0.76-0.89			Multi-pathway
BRATS 2019				FCNN
BRATS 2016 (T1c)	97.6	86.6	91.08	PGGAN
BONBID-HIE (Proposed)	99.91	90.89	93.5	LPR
	99.81	90.79	0.836	NHC
	99.88	90.86	0.906	LPL

## Data Availability

The data and supportive information are available within the article.

## LIST OF ABBREVIATIONS

RNN-AHF: Rough Neural Network and Anti-Homomorphic Filter
HIE: Hypoxic Ischemic Encephalopathy
HF: Homomorphic Filter
AHF: Anti-Homomorphic Filter
LF_4C: Laplacian 4 Connected Filter
LF_8C: Laplacian 8 Connected Filter
GF: Gaussian Filter
BLF: Bilateral Filter
RNN: Rough Neural Network
NN: Neural Network
RST: Rough Set Theory
UA: Upper Approximation
LA: Lower Approximation
INDR: Indiscernibility Relation
INFS: Information System
LRSM: Local Redundancy Stability Measure
NBD RS: Neighborhood Rough Set
CG: Conjugate Gradient
LM: Levenberg Marquardt
RMLM: Reduced Memory Levenberg Marquardt
POA: Pelican Optimization
HO: Hippopotamus Optimization
PSO: Particle Swarm Optimization
